# Role of Astrocytes in the Pathophysiology of Lafora Disease and Other Glycogen Storage Disorders

**DOI:** 10.3390/cells12050722

**Published:** 2023-02-24

**Authors:** Jordi Duran

**Affiliations:** 1 Institut Químic de Sarrià (IQS), Universitat Ramon Llull (URL), 08017 Barcelona, Spain; jordi.duran@iqs.url.edu; 2 Institute for Bioengineering of Catalonia (IBEC), The Barcelona Institute of Science and Technology, 08028 Barcelona, Spain; 3 Institute for Research in Biomedicine (IRB Barcelona), The Barcelona Institute of Science and Technology, 08028 Barcelona, Spain

**Keywords:** glycogen, aggregation, Lafora disease, neuroinflammation, neurodegeneration, epilepsy

## Abstract

Lafora disease is a rare disorder caused by loss of function mutations in either the EPM2A or NHLRC1 gene. The initial symptoms of this condition are most commonly epileptic seizures, but the disease progresses rapidly with dementia, neuropsychiatric symptoms, and cognitive deterioration and has a fatal outcome within 5–10 years after onset. The hallmark of the disease is the accumulation of poorly branched glycogen in the form of aggregates known as Lafora bodies in the brain and other tissues. Several reports have demonstrated that the accumulation of this abnormal glycogen underlies all the pathologic traits of the disease. For decades, Lafora bodies were thought to accumulate exclusively in neurons. However, it was recently identified that most of these glycogen aggregates are present in astrocytes. Importantly, astrocytic Lafora bodies have been shown to contribute to pathology in Lafora disease. These results identify a primary role of astrocytes in the pathophysiology of Lafora disease and have important implications for other conditions in which glycogen abnormally accumulates in astrocytes, such as Adult Polyglucosan Body disease and the buildup of *Corpora amylacea* in aged brains.

## 1. Brain Glycogen

Cells store glucose in the form of glycogen—a branched polymer of glucose—to minimize the increase in osmotic pressure that would be associated with the accumulation of free glucose. Glycogen molecules can store up to 55,000 glucose units in a water-soluble form that can be rapidly degraded when energy is required. This polysaccharide is synthesized through the coordinated action of glycogen synthase (GS), which joins glucose units by α-1,4-glycosidic linkages, and glycogen branching enzyme (GBE), which introduces branching points via α-1,6-glycosidic linkages [1]. Similarly, glycogen is broken down by the coordinated action of glycogen phosphorylase and glycogen debranching enzyme, which digest α-1,4- and α-1,6-glycosidic linkages, respectively. In normal glycogen, branches are introduced at even intervals and this branched structure is important for its function and solubility, generating a spherical molecule that can be rapidly degraded when the cell needs glucose. In contrast, in poorly branched glycogen, long α-1,4 linked glucose chains can form single or double helices [2,3,4] that exclude water, thereby impeding the degradation of glycogen and decreasing its solubility.

Some tissues, such as skeletal muscle and particularly the liver, accumulate high amounts of glycogen (up to 2% and 8% of wet weight, respectively) [5]. Muscle glycogen provides energy for contraction during intense exercise, while liver glycogen is mobilized during fasting periods to produce glucose, which is then released into the blood to be used by the rest of the body. All other tissues and cell types contain glycogen, although at lower concentrations than those found in the liver and skeletal muscle. Within the brain, glycogen concentration is relatively low—about 0.1% of wet weight—and it is present mostly in astrocytes [6,7,8,9,10]. However, neurons also have an active glycogen metabolism that plays an essential role in the function of these cells [11,12].

Given the low concentration of glycogen in the brain, its role in this organ was traditionally overlooked and widely presumed to serve as an emergency reservoir for pathophysiological conditions like hypoglycemia or ischemia [13]. However, it is now clear that glycogen plays key roles in the normal functioning of the brain under physiological conditions [14,15]. Brain glycogen is mobilized as supplemental fuel when energy needs increase due to neuronal activity [16,17], and substantial deficits in learning and memory arise when its use is blocked [12,18,19,20,21]. To support neuronal function, and according to the astrocyte-neuron lactate shuttle hypothesis, astrocytes degrade glycogen to generate lactate, which they then release and which is taken up and oxidized by neurons [22]. Another simpler hypothesis is that, in situations of high-energy demand, astrocytes use glucose obtained from their own glycogen store, thereby sparing interstitial glucose for neurons [23].

## 2. Lafora Disease

Despite the key physiological role of glycogen, this polysaccharide can also participate in brain pathology. In some conditions, glycogen abnormally accumulates in the nervous tissue. Lafora disease (LD, OMIM 254780) is probably the most striking example of the consequences of the abnormal buildup of glycogen in the brain. In LD, poorly branched glycogen accumulates in the brain and other tissues in the form of aggregates known as Lafora bodies (LBs). Glycogen in LBs is not accessible to glycogen phosphorylase and glycogen debranching enzyme. Thus, it cannot be degraded and progressively accumulates. Glycogen in LBs also contains high levels of covalently bound phosphate, which had been hypothesized to participate in glycogen insolubility in LD [24]. However, it seems now clear that abnormal glycogen branching, not hyperphosphorylation, underlies glycogen insolubility in LD [25,26]. In addition to poorly branched, hyperphosphorylated glycogen, LBs contain several proteins, including GS, ubiquitin, and the autophagy adaptor p62 [27,28,29,30].

The onset of LD occurs in adolescence, in previously healthy children, normally in the form of epileptic seizures that are difficult to distinguish from idiopathic generalized epilepsies. Seizures escalate over time, together with a rapid decline in cognitive function. The patient develops severe dementia and eventually enters a vegetative state with continuous seizures. Death invariably comes 5 to 10 years after the onset as a consequence of *status epilepticus* or complications derived from neurodegeneration [31,32,33]. LD is a rare disease with an estimated prevalence of ~4 cases per million individuals in the world [31]. However, the number of undiagnosed cases might be higher, particularly in developing countries. Current treatment remains palliative, with limited success in the modulation of symptoms.

**Figure 1:**
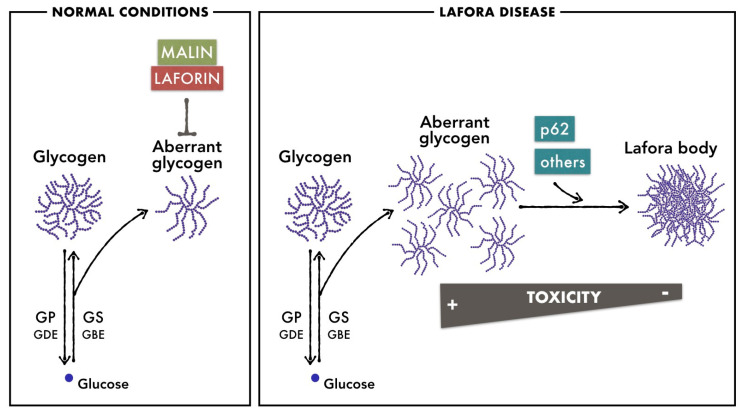
Brain glycogen metabolism in normal conditions and in LD. In normal conditions, malin and laforin prevent the accumulation of poorly branched glycogen that is generated as a side product of glycogen metabolism. In LD, due to the absence of malin or laforin, poorly branched glycogen accumulates in astrocytes and neurons. Proteins like the autophagy adaptor p62 promote its aggregation in the form of LBs to minimize the toxic consequences of its accumulation.

LD is an autosomal recessive disease caused by mutations in two genes: EPM2A, encoding laforin, a dual phosphatase that contains a carbohydrate-binding domain [34,35], and EPM2B/NHLRC1, encoding malin, an E3-ubiquitin ligase [36]. Mutations in either of these genes cause an indistinguishable disease. The exact roles of malin and laforin in glycogen metabolism are not yet fully understood, but it is widely accepted that malin uses laforin as a scaffold to bind to glycogen and ubiquitinate proteins involved in glycogen metabolism [37,38,39]. The accumulation of poorly branched glycogen in LD suggests that malin and laforin form this functional complex to regulate glycogen synthesis and prevent glycogen insolubility [40]. To minimize the toxic consequences of the accumulation of poorly branched glycogen, proteins like the autophagy adaptor p62 promote its compaction in the form of LBs [29] (Figure 1). This protective mechanism is reminiscent of the condensation of ubiquitinated, misfolded proteins into larger structures to be degraded by autophagy [41,42,43].

To study LD, several mouse models of the disease lacking laforin or malin have been generated [44,45,46,47]. These animals present similar pathophysiological phenotypes that recapitulate the human disease; i.e., they accumulate LBs in several tissues, including the brain, show a progressive neuronal loss, behavioral impairments, neuroinflammation with reactive astrocytes and microglia, altered autophagy, and increased susceptibility to epileptogenic drugs such as kainate and pentylenetetrazole [27,29,44,45,46,47,48]. In these LD models, the accumulation of LBs increases with age and the pathological phenotypes also worsen progressively as the animals age [27,28,44,49,50].

The role of LBs in the pathophysiology of LD has been unclear for many years. For instance, it was hypothesized that the primary cause of LD was an impairment in autophagy and that the accumulation of LBs was a consequence of this defect [48,51,52,53]. However, several groups, including ours, took advantage of mouse models of LD to demonstrate that excess glycogen underlies the pathology of this disease. Indeed, impeding or reducing glycogen synthesis in malin- or laforin-deficient mice prevents LB formation and prevents all the pathologic traits of the disease [27,45,54,55]. These models also showed that autophagy impairment is secondary to LB accumulation since autophagy markers are also normalized when glycogen accumulation is prevented [27,50]. Furthermore, we also used *Drosophila* and mouse models to demonstrate that forced accumulation of glycogen in neurons induces their death by apoptosis [56]. All these findings identified excessive glycogen accumulation as an inducer of neurodegeneration, and glycogen synthesis therefore became a putative target for the treatment of LD. Therapies based on the inhibition of glycogen synthesis are the focus of current research efforts [28,57,58,59,60].

## 3. Lafora Bodies in Neurons and Astrocytes

In the first description of LD in 1911, Dr. Gonzalo Rodriguez-Lafora reported the presence of LBs in neurons [61]. Until recently, it was widely believed that LBs accumulated exclusively in this cell type, and thus, all the pathologic traits of the disease were attributed to the toxic effects of neuronal LBs [31,62,63,64]. However, the premise that LBs are present exclusively in this cell population was inconsistent with the fact that, as mentioned before, brain glycogen is present mainly in astrocytes in normal conditions. It is now clear that LBs also accumulate in astrocytes. In 2011, we first reported the presence of LBs in these cells in a malin-deficient mouse model [46], but the significance of this discovery was underestimated. Several years later, we [65] and others [66] demonstrated that most LBs are present in astrocytes, particularly in regions like the hippocampus. We classified these bodies into neuronal (nLBs), and *Corpora amylacea*-like (CAL), the latter present in astrocytes, which were named this way because of their resemblance to *Corpora amylacea*, which are glycogen aggregates that accumulate in aged brains [67] (see below). Interestingly, CAL and nLBs differ not only in the cell type in which they accumulate but also in their shape and subcellular localization. CAL are polymorphic and present predominantly in astrocytic processes, and they show a patchy distribution, each patch corresponding to an individual astrocyte. In contrast, nLBs are normally present in the form of a single spherical aggregate close to the neuronal nucleus, and they resemble inclusion bodies formed by protein aggregates such as Lewy bodies [68]. The progressive accumulation of CAL and nLBs takes place in parallel, and both types of LB are already present at early stages in mouse models of LD [28,66].

**Figure 2:**
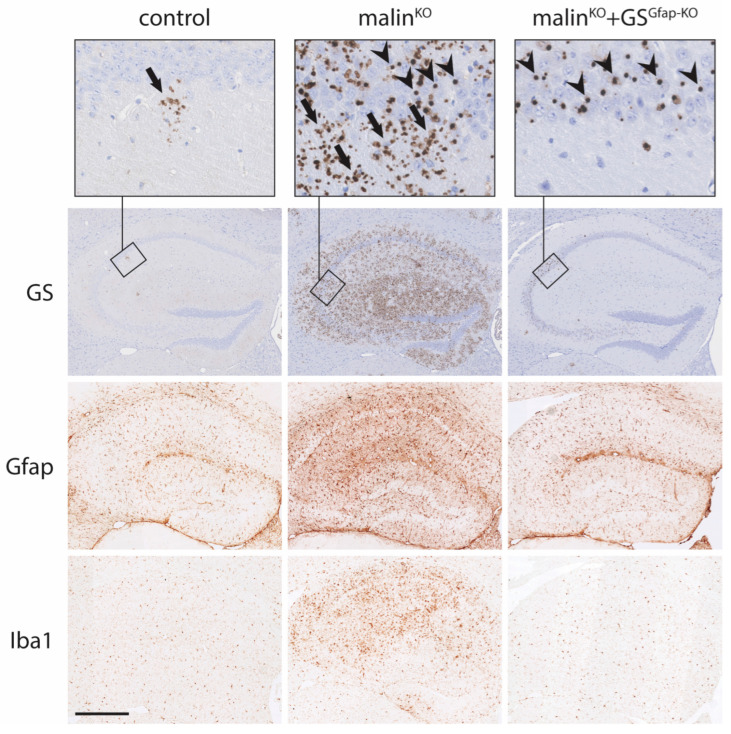
Accumulation of glycogen aggregates in neurons and astrocytes and neuroinflammation in mouse models of LD. GS, glial fibrillary acidic protein (Gfap) and ionized calcium-binding adapter molecule 1 (Iba1) immunostainings of mouse hippocampi are shown. GS immunostaining reveals the presence of few CAL aggregates in control mice. In contrast, the hippocampi of malin^KO^ mice show a conspicuous accumulation of CAL and nLBs, while the hippocampi of malin^KO^ + GS^Gfap-KO^ mice only show nLBs (although not illustrated in this summary figure, the cell types containing the aggregates have been identified in several studies by using co-staining with cell-specific markers; e.g., see [46,65,66,67]). Gfap and Iba1 immunostainings, markers of astroglia and microglia, respectively, show a prominent neuroinflammation in the brains of malin^KO^ mice that is not present in malin^KO^ + GS^Gfap-KO^ brains. Arrows: CAL, arrowheads: nLBs. Scale bar: 500 μm.

Astrocytes have been shown to have phagocytic activity and can engulf apoptotic cells [69]. Thus, LBs present in astrocytes might not have originated in astrocytes themselves, but instead, they may have a neuronal origin; i.e., proceeding from the phagocytosis of an apoptotic body derived from a dead neuron. To decipher the origin of astrocytic LBs, as well as to understand their contribution to the pathology of LD, we generated a malin-deficient mouse in which GS was specifically deleted from astrocytes (malin^KO^ + GS^Gfap-KO^ mice), thus preventing the synthesis of glycogen specifically in this cell type. The brains of these animals contained nLBs but were devoid of CAL, thereby unequivocally demonstrating that the latter originate in astrocytes [50] (Figure 2).

## 4. Role of Astrocytic LBs in the Pathophysiology of LD

The demonstration that LBs are also present in astrocytes opened up the possibility that these astrocytic LBs contribute to the pathology of LD. In fact, the quantification of CAL and nLBs showed that in brain regions like the hippocampus, CAL are clearly predominant over nLBs [65,66]. The analysis of malin^KO^+GS^Gfap-KO^ brains confirmed this quantification since the hippocampi of these mice are largely free of LBs [50] (Figure 2). Furthermore, RNA-Seq studies indicated that most of the upregulated genes in the brains of malin- and laforin-deficient mice encode pro-inflammatory mediators and that reactive glia, including astrocytes, are responsible for the expression of these inflammatory genes [70].

To understand the contribution of astrocytic LBs to the pathology of LD, the characteristic pathologic traits of the disease were analyzed in malin^KO^ + GS^Gfap-KO^ mice. Neuroinflammation is considered one of the initial determinants of LD [71]. The brains of LD mouse models present clear astrogliosis, microgliosis, and increased expression of inflammatory genes [50,70]. In contrast, the analysis of malin^KO^ + GS^Gfap-KO^ brains revealed normal levels of all these markers of neuroinflammation, thereby indicating that astrocytic LBs underlie neuroinflammation in LD [50] (Figure 2). The link between the excessive accumulation of astrocytic glycogen and neuroinflammation was further confirmed with another mouse model in which a constitutively active form of GS was expressed specifically in astrocytes. These mice, which accumulate high amounts of glycogen in astrocytes, present profound astrogliosis and microgliosis, as well as a marked increase in the expression of inflammatory genes [50]. These results confirmed that the excessive accumulation of glycogen in astrocytes induces neuroinflammation. However, the mechanism that links excessive astrocytic glycogen accumulation with neuroinflammation is currently unknown and is the focus of current research efforts.

As mentioned before, the initial symptom of LD is most commonly the presence of epileptic seizures, which worsen progressively with age. Animal models of LD reproduce this pathologic trait of the disease in the form of increased susceptibility to epileptogenic drugs like kainic acid. Astrocytes play essential roles in brain function, including the regulation of extracellular potassium and glutamate homeostasis, thus making them crucial actors in epilepsy [72]. In this regard, astrocytic glycogen has been shown to fuel potassium uptake into astrocytes, since the astrocytic sodium/potassium pump uses ATP obtained from glucose 6-phosphate originating from glycogen breakdown [73,74]. The non-clearance of extracellular potassium would result in neuronal hypersynchronization and burst firing, which would result in seizure generation and propagation. Thus, it has been suggested that alterations in glycogen metabolism contribute to the imbalance of glutamatergic and GABAergic neurotransmission associated with epileptic seizures [75]. Accordingly, it was reasonable to hypothesize that the impairment of astrocytic glycogen metabolism in LD compromises potassium uptake, which would increase excitability and thus be responsible for the epileptic phenotype of the disease. In line with this hypothesis, the presence of glycogen aggregates in astrocytes has also been described in patients with temporal lobe epilepsy [76]. Surprisingly, malin^KO^ + GS^Gfap-KO^ mice do not show a significant amelioration of susceptibility to epilepsy [50], thereby indicating that astrocytic glycogen accumulation is not the main factor responsible for the epileptic phenotype of LD. Importantly, the deletion of GS specifically in astrocytes does not increase susceptibility to epilepsy per se [19]. Thus, the epileptic phenotype of LD might be attributable to neuronal LBs, most likely to those present in GABAergic interneurons, which would impair their function and generate an imbalance of glutamatergic and GABAergic transmission. In line with this notion, we described that parvalbumin interneurons of the hippocampus accumulate LBs [46] and this buildup is accompanied by damage to GABAergic neurons in mouse models of LD [77].

In summary, astrocytic glycogen accumulation drives the neuroinflammatory phenotype of LD but not the increased susceptibility to epilepsy, which might be attributable to neuronal LBs.

## 5. *Corpora amylacea*

The accumulation of glycogen in the nervous tissue is not exclusive to LD. As mentioned before, the presence of glycogen aggregates known as *Corpora amylacea* (“starch bodies” in Latin, due to their resemblance to starch) has also been observed in aged human brains [78]. Interestingly, these aggregates accumulate to a greater extent in neurodegenerative conditions like Alzheimer’s, Parkinson’s, Huntington’s, and Pick’s diseases, as well as in patients with temporal lobe epilepsy [67,79]. Although the cellular localization of *Corpora amylacea* has been a source of debate, several articles have described their presence in astrocytes [80,81,82,83,84]. Similar aggregates progressively accumulate with age in the astrocytes of control mice [30,65,67] (Figure 2). The composition of *Corpora amylacea* greatly resembles that of LBs, consisting of insoluble, poorly branched glycogen and a minor content of protein, including GS, ubiquitin, and p62 [67]. Interestingly, these glycogen aggregates are not found in the brains of aged GS knockout mice [30]. This observation thus indicates that, as for LBs, glycogen synthesis is a prerequisite for the formation of *Corpora amylacea*. In contrast, the formation of these aggregates is enhanced in models of accelerated aging, such as the Senescence Accelerated Mouse-Prone 8 (SAMP8) mouse [65]. The overexpression of protein targeting to glycogen (PTG), an activator of GS, also resulted in an increase in the formation of these glycogen aggregates [65]. This observation suggests that an imbalance between GS activity and GBE activity favors the formation of poorly branched glycogen, which would accumulate in the form of *Corpora amylacea*-like structures (see adult polyglucosan body disease below). Strikingly, the brains of control, SAMP8, and PTG-overexpressing animals show the presence of CAL but not nLBs [65]. Therefore, the latter seem to be exclusive to LD models.

Collectively, all of points explained above suggest that the progressive accumulation of *Corpora amylacea* in the nervous system contributes to the neurological decline associated with aging [79,85]. Mutations in malin and laforin would drastically increase the rate of this process; i.e., LD could be considered an accelerated aging process with respect to the consequences of glycogen accumulation in the brain. Furthermore, the increased presence of *Corpora amylacea* in neurodegenerative conditions like Alzheimer’s and Parkinson’s disease opens up the possibility that the toxicity induced by glycogen accumulation also participates in the pathology of these disorders. Alternatively, the presence of waste elements in *Corpora amylacea* has led some authors to hypothesize that these structures are waste containers in which deleterious or residual products are isolated for later removal by the natural immune system [78,86,87].

## 6. Adult Polyglucosan Body Disease

Another rare genetic condition in which glycogen accumulates abnormally in the nervous tissue is adult polyglucosan body disease (APBD, OMIM 263570). This is an autosomal recessive neurodegenerative disorder with onset normally in the 5th or 6th decade of life and slow progression, affecting the central and peripheral nervous system with severe leukodystrophy, atrophy of the spine and medulla, and cognitive impairment [88,89,90]. The disease is caused by mutations in GBE that result in the formation of glycogen with low solubility due to the lack of branching. Consequently, there is a progressive intracellular accumulation of glycogen aggregates (the so-called polyglucosan bodies), which are similar to LBs. In fact, the term “polyglucosan body” is also used to refer to LBs and *Corpora amylacea* [78,91] APBD is caused by mutations that generate a partial loss of GBE activity (with 5–20% residual activity), the most common of which is p.Y329S, found in patients of Ashkenazi Jewish descent [92]. Other mutations in GBE cause a clinically heterogeneous disorder collectively known as glycogen storage disease IV (OMIM 232500), with hepatic and neuromuscular presentations [93]. A mouse model with the GBE mutation p.Y329S presents a phenotype that is reminiscent of APBD in humans, with the accumulation of polyglucosan bodies and neurological dysfunction [94]. This model has allowed researchers to demonstrate that, like LD, APBD can be rescued by inhibiting GS [95], again evidencing the role of glycogen accumulation in the etiopathogeny of neurodegeneration. In this regard, strategies targeting glycogen synthesis have proven effective in mouse models of both LD and APBD [59,60].

Similarly to LD, polyglucosan bodies have been reported to accumulate in astrocytes and neurons in APBD [96,97]. The exact contribution of astrocytic polyglucosan bodies to the pathophysiology of this disease is not clear. To unequivocally dissect this contribution, GS should be ablated specifically in astrocytes in the APBD mouse model, in a similar fashion as malin^KO^ + GS^Gfap-KO^ mice for LD [50]. However, after all of the considerations above, it is reasonable to hypothesize that astrocytic polyglucosan bodies participate in the pathophysiology of APBD, probably by inducing neuroinflammation in a similar fashion as LBs in LD. In fact, the presence of polyglucosan bodies in astrocytes is sufficient to cause APBD [98].

## 7. RBCK1 Deficiency

RANBP2-Type and C3HC4-Type Zinc Finger Containing 1 protein (RBCK1, also known as HOIL1) is another E3 ubiquitin ligase that is related to glycogen metabolism. Mutations in the RBCK1 gene result in polyglucosan body myopathy with or without immunodeficiency (OMIM 615895). This disease affects children, and courses with progressive proximal muscle weakness and dilated cardiomyopathy, accompanied in some cases by severe immunodeficiency and a hyperinflammatory state [99,100]. The muscle and the heart of these patients show extensive polyglucosan body accumulation [100]. Although the disease is primarily a skeletal and cardiac myopathy, a mouse model of this condition also shows the presence of profuse aggregates of poorly branched glycogen in the nervous tissue, especially in the hippocampus, cerebellum, and spinal cord [101]. Interestingly, these polyglucosan bodies are localized mainly in astrocytes, and this accumulation is accompanied by astrogliosis and microgliosis, again linking neuroinflammation to the excessive accumulation of glycogen in astrocytes [101]. These observations indicate that the accumulation of polyglucosan bodies in the nervous tissue might also play a role in the pathophysiology of human RBCK1 deficiency. As with LD and APBD, the inhibition of glycogen synthesis rescues the pathological traits of the mouse model of RBCK1 deficiency, once again demonstrating that glycogen synthesis is a prerequisite for the formation of the polyglucosan bodies that underlie the disease [101]. Of note, a recent report has shown that the substrate of RBCK1 ubiquitination is not a protein but glycogen itself. More specifically, RBCK1 targets unbranched glucosaccharides, participating in a mechanism aimed at preventing polyglucosan body accumulation [102]. Thus, different E3 ubiquitin ligases (malin and RBCK1) are involved in preventing abnormal glycogen accumulation. Interestingly, ubiquitin is present in glycogen aggregates both in the absence of malin and of RBCK1 [48,103]. The genuine substrate of malin is still not clear, but the restoration of malin expression in a malin-deficient mouse model results in the degradation of the accumulated GS and laforin [104], thereby indicating that these two proteins are targets of malin ubiquitination. These experiments also showed that, once LBs have accumulated in the CNS, malin restoration is not able to promote their removal. This observation thus indicates that the role of malin is related to preventing the accumulation of abnormal glycogen (Figure 2) rather than eliminating it after its accumulation.

## 8. Concluding Remarks

Comparison of the pathology of LD, APBD, and RBCK1 deficiency opens up a number of questions. Why do LD and RBCK1 deficiency affect children while APBD affects adults? Why are the neurological presentations so different between the three diseases? Do LBs, *Corpora amylacea* and the polyglucosan bodies that accumulate in APBD have any distinguishing features amongst them that would offer insights into the differences among glycogen storage diseases? The observation that APBD patients do not present epilepsy reinforces the idea that the epileptic phenotype of LD is due to the accumulation of LBs in GABAergic interneurons. But then, if the malin-laforin complex, GBE, and RBCK1 are all important to prevent the accumulation of abnormal glycogen, why does the lack of one or the other result in the accumulation of polyglucosan bodies with different cell type-specificity?

Over recent years, the role of astrocytic dysfunction in neurodegenerative diseases previously thought to have an exclusively neuronal origin is becoming apparent. LD is not only an example of such diseases but also one in which the pathology is due in part to a defect originated primarily in astrocytes.

In conclusion, the study of LD has allowed the identification of the toxic consequences of the excessive accumulation of glycogen in astrocytes, a process that plays a key role in the pathophysiology of LD. This pathologic mechanism might have important implications for other conditions in which glycogen abnormally accumulates in astrocytes, such as in other rare conditions like APBD and RBCK1 deficiency, and more common neurodegenerative conditions like Alzheimer’s, Parkinson’s, Huntington’s, and Pick’s diseases, or even during normal aging. Further research is needed to understand the molecular mechanisms that link excess glycogen in astrocytes with neuroinflammation.
